# P-233. Catheter-Related Bloodstream Infection (CRBSI) and Catheter-Associated Bloodstream Infection (CABSI) Outside of the Intensive Care Unit (ICU): Preliminary Insights from an Italian Monocentric Study

**DOI:** 10.1093/ofid/ofae631.437

**Published:** 2025-01-29

**Authors:** Giovanni Scaglione, Marta Colaneri, Fabio Borgonovo, Camilla Genovese, Lucia Galli, Rebecca Fattore, Monica Schiavini, Federico Fassio, alba Taino, Francesco Casella, Maria Calloni, Martina Offer, Antonio Gidaro, Andrea Gori, Antonella Foschi

**Affiliations:** Division of Infectious Diseases, Luigi Sacco Hospital, University of Milan, Milan, Italy, Milano, Lombardia, Italy; Department of Biomedical and Clinical Sciences, University of Milan, Milan, Italy, Milano, Lombardia, Italy; Division of Infectious Diseases, Luigi Sacco Hospital, University of Milan, Milan, Italy, Milano, Lombardia, Italy; Division of Infectious Diseases, Luigi Sacco Hospital, University of Milan, Milan, Italy, Milano, Lombardia, Italy; Division of Infectious Diseases, Luigi Sacco Hospital, University of Milan, Milan, Italy, Milano, Lombardia, Italy; Division of Infectious Diseases, Luigi Sacco Hospital, University of Milan, Milan, Italy, Milano, Lombardia, Italy; Division of Infectious Diseases, Luigi Sacco Hospital, University of Milan, Milan, Italy, Milano, Lombardia, Italy; Department of Public Health, Experimental and Forensic Medicine, Unit of Biostatistics and Clinical Epidemiology, University of Pavia, Pavia, Italy, Pavia, Lombardia, Italy; Division of Internal Medicine, Luigi Sacco Hospital, University of Milan, Milan, Italy, Milan, Lombardia, Italy; Division of Internal Medicine, Luigi Sacco Hospital, University of Milan, Milan, Italy, Milan, Lombardia, Italy; Division of Internal Medicine, Luigi Sacco Hospital, University of Milan, Milan, Italy, Milan, Lombardia, Italy; Department of Biomedical and Clinical Sciences, University of Milan, Milan, Italy, Milano, Lombardia, Italy; Division of Internal Medicine, Luigi Sacco Hospital, University of Milan, Milan, Italy, Milan, Lombardia, Italy; Infectious Diseases and Immunopathology, Department of Clinical Sciences, Università di Milano, Luigi Sacco Hospital, Milan, Italy, Milano, Lombardia, Italy; Division of Infectious Diseases, Luigi Sacco Hospital, University of Milan, Milan, Italy, Milano, Lombardia, Italy

## Abstract

**Background:**

Vascular access device (VAD) placement is an increasingly performed procedure linked to hospital-acquired bloodstream infections.

VADs comprise peripheral venous catheters (mid-tight, mini-midline, midline), centrally (CICC, FICC), and peripherally (PICC) inserted central venous catheters. CRBSI and CABSI definitions are often improperly used, and data beyond ICU settings are scarce.

This study investigates risk factors and the prevalence and incidence of CABSI and CRBSI episodes in non-ICU wards.

**Table 1.**
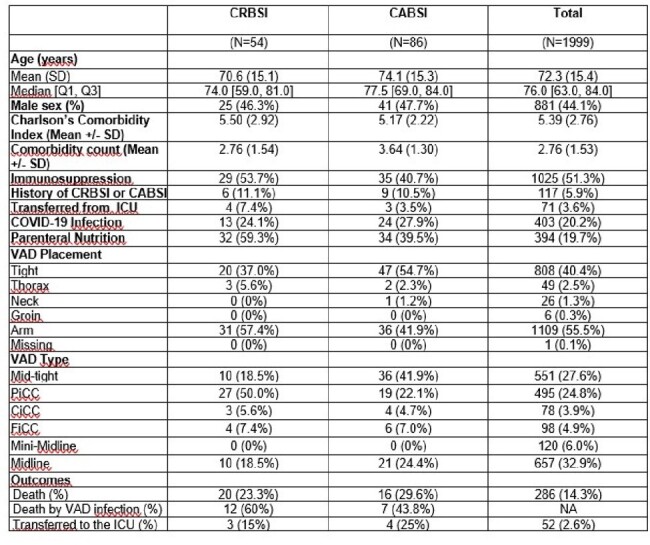
Characteristics and outcomes of the study population Immunosuppression: active onco-hematological, rheumatological disorder, solid organ transplant, hematopoietic stem-cell transplantation, or recent (<1 year) treatment, with chemotherapy or prolonged immunosuppressive treatment of any of these conditions. PiCC: Peripherally inserted Central venous Catheter, CiCC: Centrally inserted Central venous Catheter, FiCC: Femorally inserted Central venous Catheter

**Methods:**

This retrospective, observational study (January 2021- December 2023) was conducted in Luigi Sacco Hospital, Milan. Clinical and demographic data of hospitalized non-ICU patients undergone VAD placement were retrieved from medical records.

Continuous variables were described with median and interquartile [IQR], and categorical variables with counts and percentiles. A multivariable logistic regression accounting for patients’ and VADs’ characteristics was performed.

**Figure 1.**
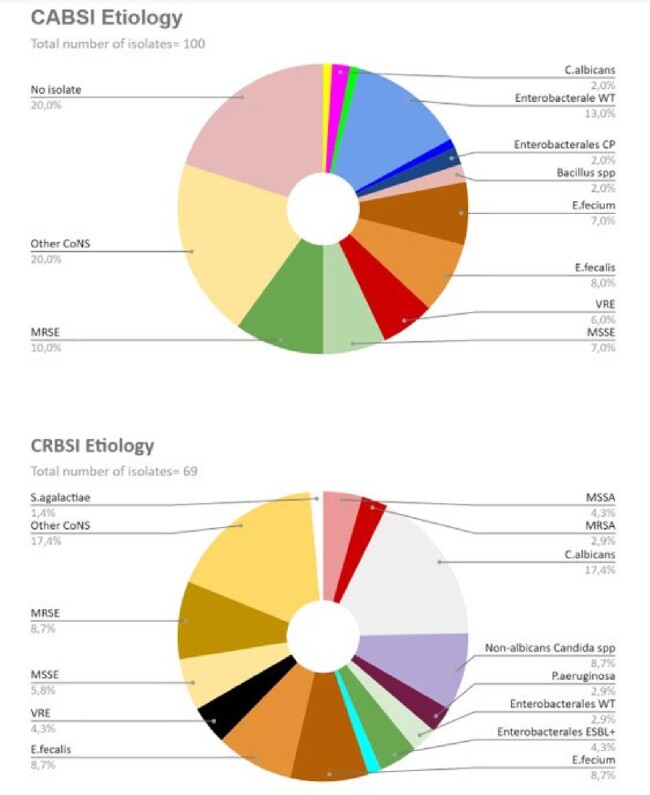
Microbiological data of CABSI (top) and CRBSI (bottom) episodes MSSA: Methicillin-sensible S.aureus, MRSA: Methicillin-resistant S.aureus, MSSE: Methicillin-sensible S.epidermidis, MRSE: Methicillin-resistant S.epidermidis, Enterobacterales WT: sensible to III gen. cefalosporins, CP: carbapenemase-producing, VRE: Vancomycin-resistant Enterococci, CoNS: Coagulase-negative Staphylococci

**Results:**

A total of 1999 VAD placements were included, patients’ characteristics and outcomes are outlined in Table 1.

Peripheral devices were 66.4%, and 54 CRBSI and 86 CABSI events were identified, according to international guidelines. CABSI and CRBSI displayed prevalence rates of 0.049 and 0.033, and incidences of 1.85 and 1.16 episodes per 1000 catheter days respectively. Parenteral nutrition was the only factor associated with CABSI and CRBSI events (p< 0.0001).

Coagulase-negative *Staphylococci* (CoNS) were isolated in 37.0% of CABSI, and 31.9% of CRBSI episodes, most frequently *S*.*epidermidis* (16%), followed by *E.fecium*, and *E.fecalis*, resistant to first-line agents in 59.2%, 35.3%, and 18.8% of cases respectively. *Candida* spp. was isolated in 12.4% of cases, and polymicrobial etiology was 17.9%.

Pathogens’ prevalence and susceptibility profiles are depicted in Figure 1.

**Conclusion:**

We observed low CRBSI and CABSI incidences in non-ICU wards. CoNs were most prevalent, and parenteral nutrition was associated with VAD-related infections, as already described.

Despite the non-ICU setting, Candida, drug-resistant GNB, and polymicrobial infection rates were alarming, stressing the importance of correct VAD management in non-ICU wards to prevent difficult-to-treat infections.

**Disclosures:**

**All Authors**: No reported disclosures

